# The modified Rowland Universal Dementia Assessment Scale for dementia screening in the Peruvian population

**DOI:** 10.1590/1980-5764-DN-2025-0377

**Published:** 2025-12-19

**Authors:** Nilton Custodio, Marcio Soto-Añari, Rosa Montesinos, Marco Malaga, Belen Custodio, Diego Chambergo-Michilot, Diego Bustamante-Paytan, Zadith Yauri, Katherine Agüero, Graciet Verastegui, Serggio Lanata, Christoper Butler, Giuseppe Tosto

**Affiliations:** 1 Instituto Peruano de Neurociencias, Unidad de Diagnóstico de Deterioro Cognitivo y Prevención de Demencia, Lince, Lima, Perú.; 2 Universidad Católica San Pablo, Departamento de Psicología, Arequipa, Perú.; 3 Instituto de Neurociencia Cognitiva, Unidad de Investigación, Arequipa, Perú.; 4 Universidad de San Martín de Porres, Facultad de Medicina, Centro de Investigación del Envejecimiento, Lima, Perú.; 5 Equilibria, Unidad de Investigación, Surco, Lima, Perú.; 6 University of California, San Francisco CA, United States of America.; 7 Weill Institute for Neurosciences, Memory and Aging Center, San Francisco CA, United States of America.; 8 Imperial College London, Department of Brain Sciences, London, UK.; 9 Columbia University, Taub Institute for Research on Alzheimer’s Disease and the Aging Brain, College of Physicians and Surgeons, New York NY, United States of America.

**Keywords:** Dementia, Latin America, Mental Status and Dementia Tests, Alzheimer Disease, Demencia, América Latina, Pruebas de Estado Mental y Demencia, Enfermedad de Alzheimer

## Abstract

Dementia cases are rising globally, particularly in low- and middle-income countries like those in Latin America.

**Objective:**

The aim of the study was to enhance diagnostic accuracy in diverse populations; modifications to brief cognitive tools may be necessary.

**Methods:**

This cross-sectional study involved 197 participants who underwent neurocognitive assessments with both the Peruvian version of the Rowland Universal Dementia Assessment Scale (RUDAS-PE) and a modified version of RUDAS-PE (mRUDAS-PE). Statistical analyses, including chi-square tests and receiver-operator curves, were used to compare the diagnostic performance of the original and modified RUDAS.

**Results:**

The mRUDAS-PE showed improved performance in the visuospatial construction domain, with more participants achieving top scores, especially among controls and Alzheimer’s disease patients. The judgment domain also yielded higher scores for controls. Only the judgment domain modifications do not change the diagnostic accuracy.

**Conclusion:**

Judgment modifications could be considered to improve the diagnostic performance of RUDAS-PE. Further testing in populations with different educational levels from rural areas is needed to assess their broader impact.

## RESUMEN.

Los casos de demencia están aumentando a nivel global, particularmente en países de bajos y medianos ingresos como América Latina.

**Objetivo:**

Para realzar la exactitud diagnóstica en poblaciones diversas, modificaciones a las pruebas cognitivas breves pueden ser necesarias.

**Métodos:**

Este estudio transversal incluyó 197 participantes quienes fueron sometidos a una evaluación neurocognitiva con la versión peruana del RUDAS (RUDAS-PE) y una versión modificada (RUDAS-PE modificado). Los análisis estadísticos, incluyendo pruebas de chi cuadrado y curvas ROC, fueron usadas para comparar el rendimiento diagnóstico de ambas versiones del RUDAS.

**Resultados:**

El RUDAS-PE modificado mostró buen rendimiento en el dominio de construcción visuo-espacial, con más participantes alcanzando los puntajes altos, especialmente los controles y pacientes con enfermedad de Alzheimer. El dominio de juicio también mostró altos puntajes en los controles. Solo las modificaciones del juicio no cambiaron la exactitud diagnóstica.

**Conclusión:**

Modificaciones en el dominio del juicio deben ser consideradas para mejorar el rendimiento diagnóstico de RUDAS-PE. Se necesitan investigaciones adicionales en poblaciones con diferentes niveles educativos de las zonas rurales para evaluar su real impacto.

## INTRODUCTION

 Dementia cases worldwide have risen exponentially due to the aging of the population^1^, most of these located in low- to middle-income countries^2^. By 2050, it is projected that people living with dementia will increase to 150 million^3^. Regions like Latin America and the Caribbean (LAC) have experienced an increase in the prevalence of dementia over the last decade^4^, leading to address this public health problem urgently^5^. 

 Numerous strategies emphasize earlier diagnosis and intervention^6,7^, in order to delay the progression of the disease. However, due to the heterogeneity of signs and symptoms, diagnosis can be challenging, leading to a low rate of detection by primary care physicians^8,9^, particularly in the early stages. In such a context, the use of brief cognitive tests (BCT) to identify potential dementia cases has been proposed as an effective strategy^10^, given the poor availability of diagnostic resources like biomarkers and imaging^11^. 

 Most of the BCTs used in LAC countries were developed without considering factors such as education level or culture^12,13^, which could lead to biased results and incorrect diagnoses of cognitive decline. With the publication and validation of the Rowland Universal Dementia Assessment Scale (RUDAS), these factors were addressed better compared to others commonly used in BCT^14^. However, recently, some studies have shown some issues. In patients with limited (<7 years) or no formal education, the visuospatial construction, praxis, and judgment items may be affected^15-17^. Also, it is suspected that educational level influences cube drawing ability due to increased frustration and perceived image ambiguity^18,19^. In addition, the judgment item requires knowledge of modern street crossings, which, in rural areas like those located in LAC countries are absent. Moreover, this item leaves scoring open to the evaluator’s interpretation^17,20,21^. 

 In order to evaluate the modified version of RUDAS performance in populations with low levels of education, in the first phase we will evaluate it in urban populations with medium and high levels of education, while in the second phase we will administer the updated version to populations with less than 6 years of education, including illiterate individuals. Thus, we aimed to evaluate the performance of a modified version of the RUDAS (mRUDAS-PE) in participants with a middle-level education in a memory clinic setting by replacing visuospatial construction and judgment items with two more culturally broad questions. We then compared the performance with the original version in detecting Alzheimer’s disease (AD) and frontotemporal dementia (FTD). 

## METHODS

### Study design and participants

 We included 197 participants (110 controls and 87 patients with dementia: 54 with AD, 20 with FTD, and 13 with other dementias) who were recruited consecutively in the Cognitive Impairment and Dementia Prevention Unit of the Peruvian Institute of Neurosciences (IPN) in Lima, Peru, between August 2021 and September 2023. Exclusion criteria included a prior history of head trauma resulting in loss of consciousness, diagnosis of epilepsy, previous history of stroke, or inability to undergo magnetic resonance imaging (MRI) due to metal implants or severe claustrophobia. In addition, we excluded 31 participants with any abnormal findings on MRI (infarcts: 10; white matter hyperintensities with Fazekas score ³2: 6; probable cerebral amyloid angiopathy: 5; granuloma: 4; traumatic brain injury: 3; hemorrhage: 2; and brain tumor: 1) deemed by the investigators to confound the results of neurocognitive testing or the MRI visual rating scale scores. 

### Procedures

#### Neurocognitive assessment

 All participants were evaluated by neurologists and geriatricians with experience in dementia. Additionally, all patients underwent standardized neuropsychological testing by two experienced neuropsychologists, and a Clinical Dementia Rating score was obtained^22,23^. 

#### Peruvian version of the Rowland Universal Dementia Assessment Scale

 The RUDAS is a simple tool, administered within 10 min and comprised of six components (memory, visuospatial orientation, visuospatial praxis or construction, motor praxis, judgment, and language). The RUDAS has a maximum score of 30, where a lower score denotes poor cognitive performance^24^. It has been validated in rural and urban Peruvians with varying levels of education^17,25,26^. Neurologists or geriatricians administered this version of the participant’s first evaluation. It was administered, such as screening by neurologists or geriatricians in the first contact with participants. 

#### Modified Peruvian version of the Rowland Universal Dementia Assessment Scale

**Figure 1:**
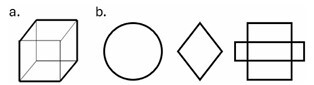
(a) The original visuospatial construction domain on the original Peruvian version of the Rowland Universal Dementia Assessment Scale (RUDAS-PE) and (b) the modified visuospatial construction domain on the modified Peruvian version of the Rowland Universal Dementia Assessment Scale (mRUDAS-PE).

 We modified two domains of RUDAS-PE: visuospatial construction and judgment. We did not alter the score for either domain. In the visuospatial construction domain, we replaced the cube drawing exercise (worth three points) with drawing three different, progressively difficult, figures: a circle, a rhombus, and an overlapping rectangle. Each of these gave one point (Figure 1). For the judgment domain, we replaced the problem of crossing the street in transit, which is scored up to 4 depending on the evaluator’s interpretation of the response, with two questions, each worth two points: what the difference between a lie and an error is, and how to locate a friend in a new city. It was administered by neuropsychologists in the first order during the comprehensive neuropsychological evaluation, blind to the screening phase. 

### Neuropsychological battery

 We used the neuropsychological battery Uniform Data Set of the National Alzheimer’s Coordinating Center (NACC) (UDS, NB 3. 0)^27^ that assesses episodic memory, processing speed, executive function, language, and construction abilities, including immediate recall of a short story (Craft Story 21 Recall), free recall of the Benson complex figure copy, number span test (forward and backward), categorical fluency (animals and vegetables), Trail Making Test A and B, delayed recall of a short story (Craft Story 21 Recall), delayed recall of the Benson complex figure copy, multilingual naming test, and verbal fluency (F and L letters). 

#### Clinical classification

 The dementia diagnosis was made according to the Diagnostic and Statistical Manual of Mental Disorders-5 criteria^22^. The AD group consisted of patients with a diagnosis of typical AD according to the published criteria from McKhann et al.^28^, and the patients with suspected FTD were diagnosed using criteria for behavioral variant frontotemporal dementia (bvFTD)^29^. The dementia severity was established using the Clinical Dementia Rating (CDR) criteria. In this study, only cases of dementia with AD (CDR of 1 or 2) were included. The CDR was applied to both study subjects and their caregivers/companions. Case diagnoses were resolved by consensus between neurologists, geriatricians, neuro-rehabilitation specialists, and neuropsychologists. 

### Statistical analyses

 We describe the demographic characteristics of cognitive groups using central tendency and frequency measures. We divided patients by diagnostic group and item and compared the proportion of participants with each score for the original and modified versions of the test. We used a χ2 test with a p-value cut-off of 0.05. Cronbach’s alpha coefficient was used to calculate homogeneity and internal consistency. The concurrent validity was assessed by determining Spearman’s rank correlation coefficient of mRUDAS-PE with Mini Mental State Examination (MMSE), Pfeffer Functionality Activities Questionnaire (PFAQ), and CDR; similarly, correlations between mRUDAS-PE and each cognitive domain were analyzed using Spearman’s correlation coefficients. Correlations were classified as very weak (0.0–0.25), weak (0.26–0.50), moderate to strong (0.51–0.75), or very strong to perfect (0.76–1.0). 

 Additionally, we used a receiver-operator curve to assess the diagnostic accuracy of the RUDAS-PE and the modified RUDAS-PE for the diagnosis of AD versus controls, FTD versus controls, and all dementia versus controls. We report the area under the curve (AUC). 

### Ethical considerations

 The research was conducted following the ethical standards of the Helsinki Declaration. The study was approved by the Committee for Medical and Health Research Ethics, Hospital Nacional Docente Madre-Niño-HONADOMANI "San Bartolomé" (10360-18). All participants participated voluntarily in the study and provided written informed consent. 

## RESULTS

### Participants

 We included a total of 197 participants, 110 controls and 87 patients with dementia: 54 with AD, 20 with bvFTD, and 13 with other dementias. Mean age was 70 [standard deviation (SD=7.8)] and mean education was 14.2 years (SD=4.1). By diagnosis, the mean years of education were 15.4 (SD=3.4) for cognitively normal patients, 13.5 (SD=3.28) for bvFTD patients, 12.4 (SD=4.97) for AD patients, and 13.1 (SD=4.01) for all other dementias (p<0.001) (Table 1). 

**Table 1:** Demographic characteristics. Demographics for the total sample overall (N=197) and split by control (N=110), Alzheimer’s disease (N=54), frontotemporal dementia (N=54), and other dementias (N=13) according to age and years of education.

		Control (N=110)	FTD (N=20)	AD (N=54)	Other dem (N=13)	Total (N=197)	p value
Age	Median (Q1, Q3)	67 (64, 72)	67 (59.5, 74)	76 (71.25, 80)	79 (74, 84)	70 (65, 76)	<0.001
	Range	60–80	50–80	56–91	59–92	50–92	
Years of education	Median (Q1, Q3)	16 (13, 17)	14.5 (11, 16)	12 (11, 16)	13 (11, 16)	15 (11, 16)	<0.001
	Range	6–25	5–18	1–26	3–18	1–26	

Abbreviations: FTD, frontotemporal dementia; AD, Alzheimer’s disease; dem, dementias.

### Visuo-spatial construction domain

**Figure 2:**
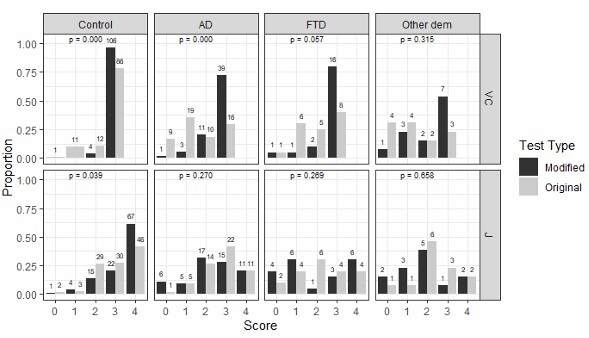
Comparative analysis between visuospatial construction (VC) and judgment (J) performance of participants, controls, and dementia on the original Peruvian version of the Rowland Universal Dementia Assessment Scale (RUDAS-PE) and the modified visuospatial construction domain on the modified Peruvian version of the Rowland Universal Dementia Assessment Scale (mRUDAS-PE).

Abbreviations: AD, Alzheimer’s disease; FTD, frontotemporal dementia; dem, dementias; VC, visuospatial construction; J, judgment.

 This domain was scored on a scale of 0–3 in both the original and modified versions. We found that a score of 3 was reached by a higher proportion of participants across diagnostic groups in the modified version compared to the original. This difference was statistically significant for controls and AD patients (p<0.000 for both groups). Over 75% of FTD and AD patients had a score of 3 in the modified version, compared to less than 50% in the original version (Figure 2). 

### Judgment domain

 Two questions were used to evaluate judgment in the original RUDAS-PE and in the modified RUDAS-PE. The total score ranged from 0 to 4 (two points per question). The proportion of patients with each score was similar for the original and modified versions of the test across all patients with a diagnosis of dementia (AD, FTD, or other). However, controls had a significantly higher proportion of high scores in the modified version compared to the original (p=0.039) (Figure 2). 

### Test diagnostic performance

 The internal consistency measured by Cronbach’s alpha coefficient on original RUDAS-PE was 0.63 [95% confidence interval (95%CI) 0.55–0.7] and on modified RUDAS-PE was 0.64 (95%CI 0.56–0.71). Strong correlations were found between the modified RUDAS-PE and MMSE (ρ=0.83; SD=0.15, 95%CI), PFAQ (ρ=0.84; SD=0.22, 95%CI), and CDR (ρ=0.85; SD=0.21, 95%CI). The modified domains (visuospatial construction and judgment) showed a weak correlation with each domain on modified RUDAS-PE (Table 2). 

**Table 2:** Correlation between total scores of the modified Peruvian version of the Rowland Universal Dementia Assessment Scale (mRUDAS-PE) and each of the cognitive domains.

	Orientation	Praxis	Visuospatial construction	Judgment	Memory	Language
Modified visuospatial construction	0.437	0.463	0.492	0.132	0.297	0.346
Modified judgment	0.233	0.274	0.341	0.312	0.379	0.325

Notes: Correlations between mRUDAS-PE and cognitive domains using Spearman’s correlation coefficients.

 We compared the diagnostic performance of the RUDAS-PE and modified RUDAS-PE tests for diagnosis of AD versus controls, FTD versus controls, and all dementias versus controls. We found that AUC was similar for all three, with an AUC of 0.95, 0.90, and 0.95, respectively, for the original RUDAS-PE, compared to an AUC of 0.95, 0.89, and 0.94 for the modified RUDAS-PE. 

 We also analyzed the performance of the modified RUDAS-PE when only the judgment domain was modified. The AUC for AD versus controls was 0.95, FTD versus controls was 0.89, and all dementias versus controls was 0.94. 

## DISCUSSION

 In this study, we modified the Peruvian version of RUDAS (RUDAS-PE) and then tested its effectiveness in diagnosing AD, FTD, and other types of dementia. Our results show that a large share of dementia participants achieved higher scores in the visuospatial construction domain, while a larger share of control patients obtained higher scores in the judgment domain, with no significant differences among dementia participants. However, when assessing diagnostic capacity, it remained more similar when the judgment domain was modified, but not the visuospatial construction domain. 

 Taking this into account, in our modified version in the judgment domain, we attempted to assess more perceptual characteristics (differences between lie and error) and functional aspects (looking for a friend). We believe that our version should be more sensitive to executive dysfunction, as the original item can be linked to procedural memory^30^. Thus, it was interesting to observe no significant difference in patients affected with FTD. However, we did observe a difference in control subjects, which, although not observed in our sample, could result in higher specificity when evaluating a sample of lower educational attainment. 

 Regarding the cube item, a higher score was achieved in control patients when applying the modified version. Taking this into account, we hypothesize that this could be related to the educational factors. In previous research, the use of 2D figures has been shown to be more appropriate in low-education populations^31^. However, these modifications could result in poor detection of preclinical neurodegeneration, where the impairment can be purely visuospatial^32^. 

 Based on our results, it could be argued that modifications to the RUDAS-PE could enhance the identification of dementia patients across diverse communities. The application of a BCT in different settings should take cultural and linguistic factors into account to improve the effectiveness of cognitive decline screening^33^. Although the RUDAS has been validated in diverse populations and has shown good performance in distinguishing dementia patients with minimal cultural and linguistic bias^34^, further studies are needed that focus specifically on low- and middle-income countries’ cultural settings and ethnic groups to better understand the influence of these factors. Furthermore, the use of guidelines for cultural adaptations in this population should be considered to improve validation procedures, like in another screening test^35^. 

### Limitations

 Having conducted this study in a population with a medium to high educational level, the scores of the mRUDAS-PE may reflect some bias linked to cognitive reserve^36^. Cognitive reserve mechanisms could compensate for performance and show a ceiling effect. It is pertinent to analyze the behavior of the proposed changes in participants with low educational levels and even illiterates. Finally, BCTs have shown sensitivity for the diagnosis of dementia, so their use to classify a specific condition should be taken with caution^37^. 

 In conclusion, the modified version of RUDAS-PE showed different performance in the visuospatial construction domain and judgment domain modifications. While the diagnostic accuracy of both the original and modified RUDAS-PE remained high, with similar AUC values across all groups, the diagnostic accuracy was maintained only when the judgment modification was applied. Based on our findings, we recommend retaining the modifications to the judgment domain while reconsidering changes to the visuospatial construction domain. Additionally, we recommend expanding the sample to include individuals with low educational levels and illiterates from rural areas to better understand the impact of these modifications on dementia detection. 

## Data Availability

The authors are willing to allow the journal to review their data if requested.

## References

[1] Livingston G, Huntley J, Sommerlad A, Ames D, Ballard C, Banerjee S (2020). Dementia prevention, intervention, and care: 2020 report of the Lancet Commission. The Lancet.

[2] World Health Organization (2017). Global Action Plan on the Public Health Response to Dementia 2017–2025 [Internet].

[3] GBD 2019 Dementia Forecasting Collaborators (2022). Estimation of the global prevalence of dementia in 2019 and forecasted prevalence in 2050: an analysis for the Global Burden of Disease Study 2019. Lancet Public Health.

[4] Nitrini R, Bottino CMC, Albala C, Capuñay NSC, Ketzolan C, Rodriguez JJL (2009). Prevalence of dementia in Latin America: a collaborative study of population-based cohorts. Int Psychogeriatr.

[5] Custodio N, Wheelock A, Thumala D, Slachevsky A (2017). Dementia in Latin America: epidemiological evidence and implications for public policy. Front Aging Neurosci.

[6] Moyer VA (2014). Screening for cognitive impairment in older adults: U.S. preventive services task force recommendation statement. Ann Intern Med.

[7] Gauthier S, Rosa-Neto P, Morais J, Webster C (2021). World Alzheimer Report 2021: Journey through the Diagnosis of Dementia.

[8] Aldus CF, Arthur A, Dennington-Price A, Millac P, Richmond P, Dening T (2020). Undiagnosed dementia in primary care: a record linkage study. NIHR Journals Library.

[9] van den Dungen P, van Marwijk HW, van der Horst HE, Moll van Charante EP, Macneil Vroomen J, van de Ven PM (2012). The accuracy of family physicians’ dementia diagnoses at different stages of dementia: a systematic review. Int J Geriatr Psychiatry.

[10] Lopera F, Custodio N, Rico-Restrepo M, Allegri RF, Barrientos JD, Batres EG (2023). A task force for diagnosis and treatment of people with Alzheimer’s disease in Latin America. Front Neurol.

[11] Parra MA, Orellana P, Leon T, Victoria CG, Henriquez F, Gomez R (2023). Biomarkers for dementia in Latin American countries: Gaps and opportunities. Alzheimers Dement.

[12] Custodio N, Duque L, Montesinos R, Alva-Diaz C, Mellado M, Slachevsky A (2020). Systematic review of the diagnostic validity of brief cognitive screenings for early dementia detection in spanish-speaking adults in Latin America. Front Aging Neurosci.

[13] Custodio N, Herrera-Pérez E, Montesinos R, Lira D, Metcalf T (2020). Brief cognitive tests validated in Peru for detection of cognitive impairment A systematic mapping of the scientific literature. Dement Neuropsychol.

[14] Nielsen TR, Jørgensen K (2020). Cross-cultural dementia screening using the Rowland Universal Dementia Assessment Scale: a systematic review and meta-analysis. Int Psychogeriatr.

[15] Chaaya M, Phung TKT, El Asmar K, Atweh S, Ghusn H, Khoury RM (2016). Validation of the Arabic Rowland Universal Dementia Assessment Scale (A-RUDAS) in elderly with mild and moderate dementia. Aging Ment Health.

[16] Nielsen TR, Vogel A, Gade A, Waldemar G (2012). Cognitive testing in non-demented Turkish immigrants - comparison of the RUDAS and the MMSE. Scand J Psychol.

[17] Custodio N, Montesinos R, Diaz MM (2021). Performance of the rowland universal dementia assessment scale for the detection of mild cognitive impairment and dementia in a diverse cohort of illiterate persons from rural communities in Peru. Front Neurol.

[18] Franzen S, van den Berg E, Goudsmit M, Jurgens CK, van de Wiel L, Kalkisim Y (2020). A systematic review of neuropsychological tests for the assessment of dementia in Non-Western, low-educated or illiterate populations. J Int Neuropsychol Soc.

[19] Olszewska A, Sobkow A (2021). Can observing a Necker cube (really) make you more insightful? The evidence from objective and subjective indicators of insight. Polish Psychol Bull.

[20] Zilbershlag Y (2023). Pilot validation of a verbal practical judgement assessment (VPJ) among community-dwelling older adults in Israel: the first step toward a national standard. Dement Neuropsychol.

[21] Vale-Britto PHF, Rabin L, Spindola L, Nitrini R, Brucki SMD (2021). Assessment of judgment ability in a Brazilian sample of patients with mild cognitive impairment and dementia. Dement Neuropsychol.

[22] Sachdev PS, Blacker D, Blazer DG, Ganguli M, Jeste DV, Paulsen JS (2014). Classifying neurocognitive disorders: the DSM-5 approach. Nat Rev Neurol.

[23] Morris JC (1997). Clinical dementia rating: a reliable and valid diagnostic and staging measure for dementia of the Alzheimer type. Int Psychogeriatr.

[24] Storey JE, Rowland JTJ, Conforti DA, Dickson HG (2004). The Rowland Universal Dementia Assessment Scale (RUDAS): a multicultural cognitive assessment scale. Int Psychogeriatr.

[25] Custodio N, Montesinos R, Lira D, Herrera-Perez E, Chavez K, Hernández-Cordova G (2019). Validation of the RUDAS in Patients With a Middle-Level Education in Lima, Peru. Am J Alzheimers Dis Other Demen.

[26] Custodio N, Montesinos R, Lira D, Herrera-Perez E, Chavez K, Reynoso-Guzman W (2020). Validation of the RUDAS for the identification of dementia in illiterate and low-educated older adults in Lima, Peru. Front Neurol.

[27] Weintraub S, Besser L, Dodge HH, Teylan M, Ferris S, Goldstein FC (2018). Version 3 of the Alzheimer Disease Centers’ Neuropsychological Test Battery in the Uniform Data Set (UDS). Alzheimer Dis Assoc Disord.

[28] McKhann GM, Knopman DS, Chertkow H, Hyman BT, Jack CR, Kawas CH (2011). The diagnosis of dementia due to Alzheimer’s disease: Recommendations from the National Institute on Aging-Alzheimer’s Association workgroups on diagnostic guidelines for Alzheimer’s disease. Alzheimer’s Dementia.

[29] Rascovsky K, Hodges JR, Knopman D, Mendez MF, Kramer JH, Neuhaus J (2011). Sensitivity of revised diagnostic criteria for the behavioural variant of frontotemporal dementia. Brain.

[30] Freedberg MV, Reeves JA, Fioriti CM, Murillo J, Voss JL, Wassermann EM (2022). A direct test of competitive versus cooperative episodic–procedural network dynamics in human memory. Cerebral Cortex.

[31] Sepúlveda-Ibarra C, Chaparro FH, Marcotti A, Soto G, Slachevsky A (2023). Normalization of Rowland Universal Dementia Assessment Scale (RUDAS) in Chilean older people. Dement Neuropsychol.

[32] Rivera-Fernández C, Custodio N, Soto-Añari M (2021). Neuropsychological profile in the preclinical stages of dementia: principal component analysis approach. Dement Neuropsychol.

[33] Ng KP, Chiew HJ, Lim L, Rosa-Neto P, Kandiah N, Gauthier S (2018). The influence of language and culture on cognitive assessment tools in the diagnosis of early cognitive impairment and dementia. Expert Rev Neurother.

[34] Nielsen TR, Jorgensen K (2020). Cross-cultural dementia screening using the Rowland Universal Dementia Assessment Scale: A systematic review and meta-analysis. Int Psychogeriatr.

[35] Khan G, Mirza N, Waheed W (2022). Developing guidelines for the translation and cultural adaptation of the Montreal Cognitive Assessment: scoping review and qualitative synthesis. BJPsych Open.

[36] Pettigrew C, Soldan A (2019). Defining cognitive reserve and implications for cognitive aging. Curr Neurol Neurosci Rep.

[37] Custodio N, Herrera-Pérez E, Montesinos R, Lira D, Metcalf T (2020). Brief cognitive tests validated in Peru for detection of cognitive impairment A systematic mapping of the scientific literature. Dement Neuropsychol.

